# Occupational Safety and Hygiene of Dentists from Urban and Rural Areas in Terms of Sharp Injuries: Wound Structure, Causes of Injuries and Barriers to Reporting—Cross-Sectional Study, Poland

**DOI:** 10.3390/ijerph15081655

**Published:** 2018-08-04

**Authors:** Anna Garus-Pakowska, Mariusz Górajski, Ewelina Gaszyńska

**Affiliations:** 1 Department of Hygiene and Health Promotion, Medical University of Lodz, 90-647 Lodz, Poland; ewelina.gaszynska@umed.lodz.pl or kathig@csk.umed.lodz.pl; 2 Department of Econometrics, Faculty of Economics and Sociology, University of Lodz, 90-214 Lodz, Poland; mariusz.gorajski@uni.lodz.pl

**Keywords:** dentists, needle stick injuries, sharp injuries, occupational exposures, reporting, post-exposure prophylaxis

## Abstract

(1) Background: Frequent contact of the dentist with potentially infectious material (PIM) is undeniable. The aim of the study was to determine the frequency and type of injuries, as well as to identify barriers to reporting and barriers to the implementation of post-exposure prophylaxis (PEP) among dentists from urban and rural areas. (2) Methods: We surveyed 192 dentists using an anonymous questionnaire. (3) Results: During the 12 months preceding the survey, 63% of dentists from the village and 58.8% of dentists from the city suffered at least one superficial cut, and deep cuts 15.1% and 17.6% respectively. Contact with PIM through spitting on the conjunctiva was 58.9% and 52.1% (village vs. city). Needle stick injuries were 50.4% and fingers were affected in 48.8% cases. The causes of injuries were: inattention 54.7%, rush 27%, unpredictable behavior of the patient 19%, recapping 18.2%. Work in the countryside was associated with a 1.95-times greater chance of not reporting injuries. The distance from a hospital with antiretroviral treatment may be a barrier to the implementation of PEP. (4) Conclusion: The circumstances of the injuries and the reasons for not applying for antiretroviral treatment point to the areas of necessary dentist education in this topic.

## 1. Introduction

The specificity of the dentist’s environment means that the most common paths for transmission of pathogens are droplet path and airway. However, do not underestimate the importance of blood-borne infections because they carry a serious risk of illness [1]. Cases of patients’ infection with blood-borne viruses in connection with dental treatment have been described [2,3]. Yet, due to the frequency of contacts with patients with different serological status (most often unknown), the risk of a dentist getting infected may be higher [3,4]. According to recent epidemiological studies, it is estimated that 300,000 adults in Poland are infected with the hepatitis B (HBV) virus, 150,000–250,000 people are infected with hepatitis C (HCV) and around 20,000 people are infected with human immunodeficiency virus (HIV) [5,6]. An infected patient may be a source of infection for medical personnel. Medical personnel who work in settings with patient populations with a high prevalence of blood-borne infections, such as urban and tertiary-care hospitals have been shown to be at greater risk of occupational infections than those who work in rural or community hospitals [7]. The probability of virus transmission after an occupational exposure is dependent on the concentration of infectious virions in the implicated body fluid, the volume of infected material transferred, and the route of transmission [8]. For this reason, we wanted to determine how often dentists are wounded with sharp tools, types of injuries and what tools most often come to injuries.

In 2016, in Poland, the right to practice was 41.2 thousand dentists (as at 31 December 2016) [9]. Most Polish dentists (over 90%) work in the private sector, while the number of dental practices funded from public funds is decreasing year by year. Thus, the number of advice provided as part of dental practices is decreasing, and this decrease applies to both the village (by 5.1%) and cities (by 5.5%). Women use dental advice more often. In cities, they constituted 55.6% of all dental consultations, while in rural areas 54.1% [9]. In Poland in 2016, 87,886 employees were injured at work [10], of which were 9476 concerned health care workers and social assistance workers (10.8% of the total). There was no accident in the units in which one person works (i.e., for example in private medical and dental practices). Also, 2176 accidents occurred as a result of contact with sharp objects, however, they are not only injuries, e.g., medical personnel needles because statistics include all sharp objects, e.g., electrician’s or fitter’s tools employed in the healthcare sector [10]. There is no official register on accidents at work among Polish dentists, there is also no national register on injuries with sharp tools among dentists. The number of occupational diseases in Poland in 2016 was 2119, of which 149 were found in the Human Health and social work activities section. Among the dentists, a total of 41 occupational diseases were diagnosed, and only three were infectious diseases (HCV) [11].

Therefore, it seems very important to assess the problem of injuries among dentists because the official registers of occupational diseases and accidents at work may suggest underestimating the problem. From the point of view of epidemiological surveillance, it is reasonable to investigate the reasons for not reporting injuries and not reporting in order to implement post-exposure proceedings. From the point of view of preventive care, it may be useful to have knowledge about the causes and circumstances of injuries to implement or modify appropriate preventive measures, especially since most injuries can be prevented [12].

The aim of the study was:

- Determining the frequency and type of contacts of dentists with potentially infectious material (PIM),
- Analysis of the structure of dentists injuries with sharp tools,
- Analysis of circumstances contributing to injury,
- Identification of dentists’ attitudes towards the problem of reporting incidents of sharp injuries and implementation of post-exposure prophylaxis (PEP),
- Identification of barriers limiting the implementation of post-exposure proceedings.

## 2. Materials and Methods

This work is part of a nationwide survey on the exposure of healthcare professionals in Poland—doctors, dentists, nurses and paramedics—to infectious material. The presented part of the study concerned a group of dentists and was conducted among 267 selected dentists from all over the country. The details of the study population and recruitment were presented elsewhere [13]. Dentists received anonymous questionnaires prepared by the authors for the purposes of the study. The questionnaire section on exposure to infectious material included six general questions regarding the frequency and types of contacts of dentists with infectious material, both throughout their careers and during the last 12 months preceding the survey. Completed surveys were obtained from 192 dentists (adjusted response rate, 72%).

To determine the structure of wounds, 13 specific questions were also asked regarding the last incident of exposure to infectious material that occurred in the dentist. This part of the survey decided to fill 137 dentists. In several questions it was possible to choose more than one answer. The questions concerned:

- What kind of infectious material was the respondent exposed to? (e.g., blood, saliva),
- What part of the body was exposed? (e.g., forearm/shoulder, palm, finger, face, eyes),
- Type of exposure? (e.g., intact skin, non-intact skin, deep wound, conjunctiva of the eye, oral mucosa),
- What kind of tool caused the wound? (e.g., suture needle, syringe needle, drill, other dental tool, scalpel),
- During which operation did the injury take place? (e.g., injection, surgery, preparation of a carious cavity, or putting on a needle cover),
- What individual respondent was wearing at the time of injury? (e.g., protective clothing, gloves, face mask, visor),
- What circumstances contributed to the wounded according to the respondent? (e.g., rush, inattention, poor work organization, work load),
- What feelings accompanied the respondent after being wounded with a sharp tool? (e.g., nothing, fear),
- Did the respondent report a professional exposure? (yes, immediately, yes, but after a while, no, I forgot, no, I did not see such a need),
- Why did the respondent not report the wound and why were the post-exposure proceedings not implemented? (answers included in Section 3.5).

Independent variables such as gender, job position, job seniority, workplace (big city, small town, village), number of jobs, and subjectively perceived personal situation of respondents were tested where there was a possibility to answer one of two answers: (1) “I feel uncertain, there is the possibility of dismissal, I did not develop professionally”, and (2) “I am professionally fulfilled, I am sure of employment and further development”.

We used nonparametric tests for independence and correlation in order to study the existence of stochastic relationship between variables describing the general population. We decided to use three tests: Pearson’s chi-squared test and its modification for nominal variables based on V-Cramer statistics, and Spearman test, which also indicated the trend of that relationship. We applied multivariate logistic regression models to explain the log odds of the probability of an event. We also calculated the odds ratios (OR) to compare the probabilities of sharps injuries in several risk groups.

In order to examine what circumstances determine/increase the risk of injury and which variables reflect the feelings of the respondents after wounding, logistic regression models were built to explain the probabilities of a given circumstance during wounds and chances of occurrence of a given feeling among respondents. In the models for the set of explanatory variables, the following were selected: gender, seniority, place of work, number of jobs, situation at the workplace (I met professionally).

Statistical computations were performed using R-package version 3.4.3 [14] and Excel 2010 (IBM Corp., Armonk, NY, USA). The level of statistical significance was set at *p* ≤ 0.05.

The study protocol was approved by the Bioethics Committee of the Medical University of Lodz (Document No. RNN /163/14/KB of 11.02.2014) in full accordance with the World Medical Association Declaration of Helsinki.

## 3. Results

### 3.1. Characteristics of the Study Group

Of the 192 dentists, the vast majority were women (*p* < 0.05) which reflects the feminization of the profession across the country [9], with a seniority of more than 5 years, working in at least two workplaces, mostly in large cities. Most dentists were satisfied with the place and nature of their work. Demographic characteristics are presented in Table 1.

### 3.2. Frequency of Contacts with Blood and other Body Fluids

Every third dentist surveyed (out of 192), both working in the country and in the village (28.8% vs. 28.6%) stated that the dentist’s work is associated with quite frequent exposure to various risk factors that threaten his safety. Almost 83% of dentists (86.3% from rural areas vs. 80.7% from cities) admitted that they are exposed to PIM several times a day.

During the 12 months preceding the study, 28.8% of dentists working in rural areas and 26.9% of working in cities had at least one contact with PIM through damaged skin. At least once, 63% of dentists from villages and 58.8% of dentists from cities suffered superficial scarring and 15.1% and 17.6% were deeply injured respectively. Contact with PIM by spitting on the mucosa/conjunctiva of the eye was 58.9% and 52.1% (village vs. city). The differences were not statistically significant.

### 3.3. Structure of Injuries, Based on Responses to the Last Incident of Wounding Experienced by the Dentists

Describing the last PIM exposure incident were 137 dentists. Due to the type of PIM, 53.2% of dentists were exposed to blood and 41.4% to saliva.

Due to the type of exposure, every third dentist (32.8%) suffered a deep wound, and every fifth (19.9%) of PIM splashes on the face. At the same time, almost all respondents (99.3%) used the protective face shield and 93.4% of the gloves.

The parts of the body affected by the exposure were: finger (48.8%), eyes (16.6%), face (15.1%), palm (11.7%) and upper arm (6.8%).

Among the dentists, the most common instruments that caused an injury were a surgical needle (29.7%), a needle for injection (20.7%) and a drill (14.5%).

### 3.4. Circumstances of Injury and Feelings Accompanying Wounds

The most frequently mentioned contributing factor to injury was the so-called human factor—inattention, lack of concentration (*n* = 75, 54.7%). Quite often, rush was also indicated (*n* = 37, 27.0%) and unpredictable behavior of the patient (*n* = 26, 19.0%). Similarly, 26 people (19.0%) indicated that attempting to put on a worn needle casing that was inconsistent with health and safety regulations contributed to wounding. Dentists working in several offices 2.5-times more often indicated the cause of injury as “too many duties” (OR = 1/0.386 = 2.590, *p* < 0.1, 95% Confidence Interval (CI), 0.970–7.407). Dentists who were satisfied with their work, 6-times more often, responded that the cause of injury was “non-compliance with health and safety rules” (OR = 1/0.156 = 6.410, *p* < 0.05, 95% CI, 1.014–50.000). People with longer work experience had less chance of showing the answer “short work experience, no experience” (OR = 0.096, *p* < 0.05, 95% CI, 0.005–0.412). Women almost 3 times more often than men indicated the answer “unpredictable behavior of the patient” (OR = 2.777, *p* < 0.1, 95% CI, 0.940–7.759). The structure of the responses is shown in Table 2.

Every third dentist surveyed (29%) felt some level of fear after being wounded, but this fear was not long-lasting. Long-term/chronic fear for their future—their health—was felt by almost every tenth respondent (9.5%). Every fourth (25.5%) dentist believed that wounds are related to the profession. This answer was marked more often by people who were professionally satisfied (OR = 3.568, *p* < 0.05, 95% CI, 1.143–15.747). 16.8% did not care about injury because they “determined” that the patient was not infected (no dependence on the variables tested).

Male gender (OR = 6.171, *p* < 0.05, 95% CI, 1.166–32.829), professional satisfaction (OR = 6.135, *p* < 0.05, 95% CI, 1.148–35.714) and longer work experience (OR = 2.630, *p* < 0.05, 95% CI, 1.297–6.223) determined an increase in the chances of underestimating the fact of wounding. In addition, the longer seniority, the lower the chance of experiencing short-term fear (OR = 0.624, *p* < 0.05, 95% CI, 0.425–0.894) and long-lasting fear (OR = 0.561, *p* < 0.05, 95% CI, 0.391–0.785). Dentists working in big cities were more likely to feel fear after being hurt, but they quickly forgot about it (OR =2.525, *p* < 0.05, 95% CI, 1.093–6.25). At the same time, the longer the work experience, the greater the sense of security due to previous HBV vaccination (OR = 1.533, *p* < 0.05, 95% CI, 0.952–2.552) (Table 3).

### 3.5. Reporting and Post-Exposure Prophylaxis

Of the 192 respondents, almost 60% said they always report injuries. Definitely more often (Chi2 = 4.942, *p* = 0.026) incidents are reported by dentists working in cities than in rural areas (65.5% vs. 49.3%). Work in the countryside was associated with a 1.95-times greater chance of not reporting injuries (95% CI, 1.079–3.543).

Among 137 dentists who agreed to characterize their last exposure to infectious material, only 29.2% (*n* = 40) reported this incident (28 dentists from a large city and 12 dentists from a village/small town). Of these 28 people did it immediately, and 12 people only after some time. Most commonly reported were needle injection (37.5% in the villages and 50% in cities) (Table 4). We did not observe differences in reporting between the analyzed variables.

Among those exposed to PIM (*N* = 137), in 42 people, post-exposure proceedings (30.6%) were implemented. In the other cases, one of the most common causes (13.87%) of the lack of post-exposure follow-up was the belief that the punctures are an inseparable part of the dentist’s work, they happen frequently, and bad things “never” happen. A common response (16.84%) was the reluctance to cancel subsequent patient visits due to the lack of substitution. The decision not to implement the procedure was also influenced by the fear of long-term therapy (9.49%). Almost every tenth (8.42%) injured dentist did not attend the therapy because of the considerable distance from the hospital that has antiretroviral drugs. Among the answers marked as “other cause”, the most frequent was the reluctance to break “behavior patterns”—“co-workers who hurt themselves do not implement the proceedings, so I will not either” (6.6%). We did not find statistically significant relationships (Table 5).

## 4. Discussion

Authors of various studies point to the frequent contact of dentists with infectious material. In a Scottish study, 14% of dentists suffered from an acute wound during the 12 months preceding the study, and 28% in Australia [15,16]. Over the past year, about 60% of dentists have suffered superficial wounds, and 16% of dentists have suffered deep cuts. At the same time, about 56% of dentists had mucosal or conjunctival exposures. These data are closer to other results obtained in Poland by Gańczak et al. [17], in which in the year preceding the study, 87% of dentists had an acute tool, and 39% had at least one exposure through mucous membranes. Personal protective equipment is to protect the physician against contact with infectious material and thus reduce the risk of transmission of infection. Unfortunately, these universal principles are not always respected [18,19]. In our study, we did not find any negligence in the use of face shields, while dentists did not always use protective gloves. Unfortunately sometimes in Poland, the medical staff does not understand the principles of hand hygiene and personal protection. They use gloves as a substitute of hand hygiene—and not as an element of protection against microbes [20,21]. Here we find the need for increased education.

Despite the use of many sharp tools in everyday work, dentists most often suffered from needle injuries, which confirms previous research [16,22,23].

Pasha L. et al. and Moodley I. et al. pointed out that the finger is the most vulnerable part of the dentist’s body [22,24], which we also showed in our study. In addition, the face and eyes are exposed to splashing of PIM. Therefore, individual protection measures should be used to protect against contact with PIM as indicated above. At the same time, dentists should implement the use of safe protective equipment against injury.

Among the causes of injuries, the lack of concentration of attention and rush were most often mentioned. Every fifth dentist pointed to the attempt to put a cover on the used needle. This is a lower result than in other studies [25] but according to recommendations such behavior is definitely banned and should not happen at all. Therefore, we point to the second aspect of the necessary dentist education. We pointed out that a long work experience and excess of duties can contribute significantly to a greater risk of injury. However, we did not find any differences regarding the causes of injury depending on the workplace. Both dentists from large cities and villages pointed to similar risk factors for injuries.

Men, “professionally satisfied” dentists and dentists with longer work experience have significantly underestimated the fact of wounding. On the other hand, dentists working in the countryside more often feared for their health, and it was a long-term fear. Interestingly, work in the countryside was more likely not to report injury. Naturally, it would seem that if we are afraid of something, we would do everything to eliminate this fear. In the event of injury, the first step is to report it and assess the risk, and then to decide on further post-exposure proceedings.

In 2013, the Minister of Health in Poland implemented a regulation under which each injury caused by a sharp instrument is defined as a work-related accident. According to regulations, each such incident should be reported to a person responsible for keeping the register of work-related accidents [26]. The provisions of the Regulation do not apply to persons performing a medical profession in the form of an individual professional practice (e.g., in their own office). In such cases, the decision on the type of medical devices used and the safeguards they are equipped with depends solely on these people. Similarly, they do not have to submit reports to anyone. Reporting to an institution that offers the implementation of post-exposure proceedings lies solely in the interest of the dentist concerned.

According to official statistics, only three professional infectious diseases were reported among dentists in 2017. However, many authors state that the reporting of both injuries and occupational diseases is not complete [19,27,28]. What is more, so if your profession brings satisfaction (with us 82% of respondents) and it is economically viable. Notifying a disease is, in short, unprofitable and until the employee is able to work and does what he likes. Lack of willingness to report incidents confirmed our study.

In addition, among 40 people who reported injury, 12 people did it for a certain time, and yet it is known that time in the implementation of antiretroviral therapy plays a significant role [29]. Longer work experience means a greater sense of security due to vaccination. However, it should be remembered that there are no vaccines against HCV and HIV. And here again the post-exposure procedure is quickly implemented. We have shown that dentists working in the rural areas are less likely to apply for antiretroviral treatment. We have also pointed out that a significant distance from a hospital offering antiretroviral therapy could be a barrier to the implementation of PEP.

After being wounded with a sharp tool, 15.5% of dentists did not care because they determined that the patient was not infected. In the Wicker and Rabenau study it was determined that the main reason (19.1%) for not reporting needle stick injuries was little or no perception of risk on behalf of the respondent [19]. Unfortunately, we are unable to determine exactly what these arrangements were. Have patients been tested for antibodies? Did the doctor only try to determine orally the fact of being infected? Please note that according to the rules of each patient, you should be treated as potentially infected and we can not have any guarantee that you answered our questions honestly.

As we have shown in an earlier study [13], dentists have good theoretical knowledge about infections. This study points to the frequent underestimation of exposure to PIM. The assessment of infection risk and information on PEP are other areas where educational activities should be undertaken. Due to the nature of the work, often running one-person offices, education could be conducted in the Medical Chambers, or in the form of obligatory on-line courses. During these courses, dentists could receive up-to-date information on the availability of antiretroviral therapy and also about the implementation of safety-engineered sharp devices wherever possible [30].

## 5. Conclusions

Dentists are more prone to occupational exposure because of their daily contact with the patients. This study pointed an alarming rate of underreporting of injuries. Self-employed employees are not required to report exposure. The circumstances of the injuries and the reasons for not applying for antiretroviral treatment point to the areas of necessary dentist education in this topic.

## Figures and Tables

**Table 1:** Characteristics of the study group, *N* = 192.

Study Group	Small Town and Village	Big City	Total
Gender, *n* (%)			
female	63 (86.3)	99 (83.2)	162 (84.4)
male	10 (13.7)	20 (16.8)	30 (15.6)
Total	73 (38.0)	119 (62.0)	192 (100)
Job seniority (years), *n* (%)			
<5	12 (16.4)	45 (37.8)	57 (29.7)
≥5	61 (83.6)	74 (62.2)	135 (70.3)
Total	73 (38.0)	119 (62.0)	192 (100)
Number of jobs, *n* (%)			
1	37 (50.7)	44 (37.0)	81 (42.2)
≥2	36 (49.3)	75 (63.0)	111 (57.8)
Total	73 (38.0)	119 (62.0)	192 (100)
Personal situation of respondents, *n* (%)			
“I feel uncertain, there is the possibility of dismissal, I do not develop professionally”	14 (19.2)	20 (16.8)	34 (17.7)
“I am professionally fulfilled, I am sure of employment and further development”	59 (80.8)	99 (83.2)	158 (82.3)
Total	73 (38.0)	119 (62.0)	192 (100)

*n*—number of dentists.

**Table 2:** Circumstances that dentists believe have caused injury, (*N* = 240 *).

	Total Number (%)	City (%)	Village (%)	OR (*p*-Value)
Urgent medical intervention (hemorrhage, collapse)	2 (1.4)	2 (2.3)	0 (0.0)	0 (0.52) ^ns^
Lack of team cooperation	4 (2.9)	4 (4.7)	0 (0.0)	0 (0.29) ^ns^
Psycho-physical indisposition, “I had a bad day”	8 (5.8)	5 (5.8)	3 (5.8)	1.01 (1) ^ns^
Rush	37 (27.0)	22 (25.6)	15 (29.4)	1.21 (0.69) ^ns^
Inattention	75 (54.7)	47 (54.7)	28 (54.9)	1.01 (1) ^ns^
Too many duties, too much workload	19 (13.9) ^c^	9 (10.5)	10 (19.6)	2.07 (0.11) ^ns^
Bad work organization	6 (4.4)	5 (5.8)	1 (2.0)	0.33 (0.27) ^ns^
Recapping	25 (18.2)	16 (18.6)	9 (17.6)	0.93 (0.63) ^ns^
Lack of compliance with health and safety rules	5 (3.6) ^d^	5 (5.8)	0 (0.0)	0 (0.15) ^ns^
During the procedure, “I was thinking elsewhere”	3 (2.2)	2 (2.3)	1 (2.0)	0.84 (0.68) ^ns^
Short seniority, lack of work experience	8 (5.8) ^b^	6 (7.0)	2 (3.9)	0.54 (0.37) ^ns^
Unpredictable behavior of the patient	26 (19.0) ^a^	16 (18.6)	10 (19.6)	1.1 (0.52) ^ns^
It is difficult to indicate the circumstances of the wound, this is an inseparable part of the work	22 (16.0)	14 (16.3)	8 (15.7)	0.95 (0.56) ^ns^

* *N* = 240, respondents could choose more than 1 answer; ^a^ a gender-related relationship; ^b^ dependence relevant to seniority; ^c^ significant dependence on the number of jobs; ^d^ dependence significant in relation to the variable “I am fulfilled professionally”; ^ns^ not statistically significant.

**Table 3:** Feelings felt by respondents after injury, (*N* = 145 *).

	Total Number (%)	City (%)	Village (%)	OR (*p*-Value)
Nothing special, it is an inseparable part of my work	35 (25.5) ^c^	20 (23.3)	15 (29.4)	ns
Fear but I quickly forgot about it	40 (29.2) ^b^	31 (36.0)	9 (17.6)	*p* < 0.05, OR = 0.396, 95% CI 0.16–0.915
Long-term fear, panic, “what will happen next?”	13 (9.5) ^b^	4 (4.7)	9 (17.6)	*p* < 0.01, OR = 5.566, 95% CI 1.634–22.447
I did not care because I was vaccinated	16 (11.7) ^b^	11 (12.8)	5 (9.8)	ns
I did not care because I determined that the patient is not infected HIV/HBV/HCV	23 (16.8)	13 (15.1)	10 (19.6)	ns
I did not care because I had been hurt many times in similar circumstances and nothing bad happened	8 (5.8) ^a,b,c^	4 (4.7)	4 (7.8)	ns
Others	10 (7.3)	6 (7.0)	4 (7.8)	ns

* *N* = 145, respondents could choose more than 1 answer; ns—not statistically significant; 95% CI—95% Confidence Interval; ^a^ a gender-related relationship; ^b^ dependence relevant to seniority; ^c^ dependence significant in relation to the variable “I am fulfilled professionally”.

**Table 4:** The structure of injury reports depending on the type of tool and the dentist’s workplace, *N* = 137 injuries and *n* = 40 reported.

Workplace	Instrument of Injuries	Reporting *n* (%)		Total (*n*)
		No	Yes	
Village and small town	Suture needle	14 (70.0)	6 (30.0)	20
	Syringe needle	5 (62.5)	3 (37.5)	8
	Dental drill	4 (80.0)	1 (20.0)	5
	others	16 (88.9)	2 (11.1)	18
Big city	Suture needle	1 (69.6)	7 (30.4)	23
	Syringe needle	10 (50.0)	10 (50.0)	20
	Dental drill	5 (71.4)	2 (28.6)	7
	others	27 (75.0)	9 (25.0)	36
Total	Suture needle	30 (69.8)	13 (30.2)	43
	Syringe needle	15 (53.6)	13 (46.4)	28
	Dental drill	9 (75.0)	3 (25.0)	12
	others	43 (79.6)	11 (20.4)	54

**Table 5:** The most common reasons for not implementing post-exposure proceedings, (*N* = 95).

	Total Number (%)	City (%)	Village (%)	OR (*p*-Value)
The hospital deciding on the implementation of the antiretroviral procedure is far from my workplace	8 (8.42)	4 (6.89)	4 (10.81)	ns
I could not leave the job (there was no one to replace me)	16 (16.84)	7 (12.07)	9 (24.32)	ns
Post-exposure proceedings are very inconvenient and long-lasting	13 (9.49))	7 (8.14)	6 (11.77)	ns
I have been hurt many times and nothing bad has happened	19 (13.87)	12 (13.95)	7 (13.73)	ns

ns—not statistically significant.
